# Identification of Immediate Early Genes in the Nervous System of Snail *Helix lucorum*

**DOI:** 10.1523/ENEURO.0416-18.2019

**Published:** 2019-05-20

**Authors:** Chuan Xu, Qian Li, Olga Efimova, Xi Jiang, Marina Petrova, Alia K. Vinarskaya, Peter Kolosov, Nikolay Aseyev, Kira Koshkareva, Victor N. Ierusalimsky, Pavel M. Balaban, Philipp Khaitovich

**Affiliations:** 1 CAS Key Laboratory of Computational Biology, Chinese Academy of Sciences-Max Planck Gesellschaft Partner Institute for Computational Biology, Shanghai Institutes for Biological Sciences, University of Chinese Academy of Sciences, Chinese Academy of Sciences, Shanghai 200031, China; 2 Skolkovo Institute of Science and Technology, Moscow 143026, Russia; 3 Institute of Higher Nervous Activity and Neurophysiology, Moscow 117485, Russia; 4 Center for Excellence in Animal Evolution and Genetics, Chinese Academy of Sciences, Kunming 650223, China; 5 Comparative Biology Laboratory, Chinese Academy of Sciences-Max Planck Gesellschaft Partner Institute for Computational Biology, Shanghai Institutes for Biological Sciences, Chinese Academy of Sciences, Shanghai 200031, China; 6 School of Life Science and Technology, ShanghaiTech University, Shanghai 200031, China

**Keywords:** snail, immediate early genes, Helix lucorum, nervous system

## Abstract

Immediate early genes (IEGs) are useful markers of neuronal activation and essential components of neuronal response. While studies of gastropods have provided many insights into the basic learning and memory mechanisms, the genome-wide assessment of IEGs has been mainly restricted to vertebrates. In this study, we identified IEGs in the terrestrial snail *Helix lucorum*. In the absence of the genome, we conducted *de novo* transcriptome assembly using reads with short and intermediate lengths cumulatively covering more than 98 billion nucleotides. Based on this assembly, we identified 37 proteins corresponding to contigs differentially expressed (DE) in either the parietal ganglia (PaG) or two giant interneurons located within the PaG of the snail in response to the neuronal stimulation. These proteins included homologues of well-known mammalian IEGs, such as *c-jun/jund*, *C/EBP*, *c-fos/fosl2*, and *Egr1*, as well as homologues of genes not yet implicated in the neuronal response.

## Significance Statement

Gastropods, which include snails and slugs, are widely used in studies of basic neuronal activity mechanisms. The first step of the transcriptional response to neuronal stimulation requires the activation of immediate early genes (IEGs). The identification of IEGs is important for the understanding of neuronal response mechanisms and for the visualization of activated neurons. However, genome-wide studies of IEGs have thus far been mainly restricted to vertebrates. Furthermore, a study of activity-regulated genes (ARGs), a gene group that includes IEGs, conducted in fruit flies did not reveal a clear overlap with vertebrate IEGs. In this study we present a transcriptome-wide study of snail IEGs, which reveals multiple homologues of well-known mammalian IEGs, as well as a number of novel IEG candidates.

## Introduction

Immediate early genes (IEGs) are the composite group of genes rapidly and transiently upregulated in neuronal cells in response to stimulation. An induction of IEGs does not require *de novo* protein synthesis and commonly occurs within an hour after the stimulation event (Fowler et al., 2011). Stimulation experiments conducted in the presence of protein synthesis inhibitors revealed hundreds of mammalian IEGs, with most works conducted in mouse brain preparations (Thompson et al., 2010; Bojovic et al., 2015; Gerstner et al., 2016) and neuronal cultures (Kim et al., 2010; Saha et al., 2011; Spiegel et al., 2014). Although different types of stimulations induce different sets of IEGs, many genes were repeatedly found in most experiments (Dahmen et al., 1997; Bepari et al., 2012; Lacar et al., 2016). Many commonly induced IEGs, including the first identified one, *c-fos*, as well as *c-jun*, *C/EBP*, and *Egr1*, function as transcription factors triggering the expression of the secondary response genes (SRGs; West and Greenberg, 2011). As well as transcription factors, neuronal IEGs encode other functional proteins, such as cytoskeletal regulators (*Arc*), growth factors (*β*-activin), metabolic enzymes (*Dusp1*), and signal transduction proteins (*Homer*; Lanahan and Worley, 1998).

While mammalian and vertebrate IEGs are relatively well characterized, less is known about IEGs in invertebrate species. Whereas several studies have been performed exploring genes involved in the long-term memory in the *Caenorhabditis elegans* (Lakhina et al., 2015; Freytag et al., 2017) and *Aplysia kurodai* (Lee et al., 2008), the genome-wide systematic investigation focusing on IEGs in invertebrates to date has been restricted to fruit flies, with two studies characterizing neuronal activity-regulated genes (ARGs). Similar to IEGs, ARGs are defined as genes induced in neurons within approximately 1 h after the stimulation, but without *de novo* protein synthesis inhibition. The first study using microarrays to assess the transcriptome alterations in heads of mutant flies after the seizure induction identified 122 genes showing rapid expression changes. These genes included fly homologs of known mammalian IEGs, such as *c-fos*, *c-jun*, *C/EBP*, and *Egr* (Guan et al., 2005). The second study used transcriptome sequencing (RNA-Seq) to look for ARGs in the fly brain and various neuron types after the activation by three stimulation protocols (Chen et al., 2016). Although the study identified known insect IEGs of *hr38* and *sr* induced in the fly brain by the three stimulation protocols, there was no detectable activation of many mammalian IEGs’ homologues, including *c-fos* and *c-jun*. Instead, largely independent sets of genes, ∼100 each, were induced by each stimulation procedure.

While no genome-wide screens for neuronal IEGs were conducted in other invertebrate species, previous studies identified a number of individual genes. Specifically, the analysis of IEGs in a sea slug *Aplysia californica*, an organism widely used in the studies of memory mechanisms, identified homologues of mammalian IEGs *c-jun*, *C/EBP*, *CREB1*, and *Egr* (Alberini et al., 1994; Sung et al., 2006; Bonnick et al., 2012; Cyriac et al., 2013). In addition, studies conducted in the terrestrial slug *Limax valentianus* identified homologues of mammalian IEGs *C/EBP* and *KLF* (Fukunaga et al., 2006). Similarly, homologues of mammalian IEGs *C/EBP*, *CREB1*, and *CREB2* were identified in the extensively studied pond snail *Lymnaea stagnalis* (Sadamoto et al., 2004, 2010; Hatakeyama et al., 2006).

Here, we conducted a broad search for neuronal IEGs in another model invertebrate species, the terrestrial snail *Helix lucorum*. The nervous system of this organism containing five pairs of neuronal ganglia and one unpaired visceral ganglion has been used in electrophysiological studies for >35 years, yielding insights into basic learning and memory mechanisms (Balaban, 1980, 2002; Balaban et al., 2015). However, the absence of the genome sequence has limited molecular studies of the neuronal response mechanisms. To overcome this limitation, we conducted the *de novo* assembly of the snail neuronal transcriptome using >943 million reads cumulatively covering >98 billion nucleotides. The following analysis yielded 37 putative snail IEGs, including homologues of well-characterized mammalian ones: *c-jun/jund*, *C/EBP*, *c-fos/fosl2*, *Egr1*, *Ier5l*, *Socs2*, and *Dusp10*.

## Materials and Methods

### Sample preparations and RNA-Seq

We conducted experiments using adult *H. lucorum taurica L.* specimens weighing 30–35 g. The snails were kept in a wet environment and fed their usual diet of pieces of lettuce. The experimental procedures were in compliance with the Guide for the Care and Use of Laboratory Animals published by the National Institutes of Health and the protocol was approved by the Ethical Committee of the Institute of Higher Nervous Activity and Neurophysiology RAS. Before the experiment, the snails were kept in the active state for at least two weeks. Details of the preparation and identification of neurons are given elsewhere (Malyshev and Balaban, 2002). Briefly, animals were cooled to 4°C and injected with isotonic MgCl_2_ before the CNS isolation to minimize pain. The central ganglia complex was surgically isolated from anesthetized snails, pinned to a silicone-elastomer (Sylgard)-coated dish, and kept in high-Ca^2+^, high-Mg^2+^ Ringer saline (80 mМ NaCl, 4 mМ KCl, 28 mМ СаСl_2_, 25 mМ MgCl_2_, and 10 mМ Trisma; pH 7.6) to suppress electrical activity of the nervous system. Such treatment blocks electrical activity in the CNS and neuromuscular connections (Balaban and Chase, 1989), thereby minimizing the dissection effect on IEGs’ expression in our experiments. The central ganglia complex was stripped of connective tissue sheath using a ﬁne forceps and scissors, ensuring the integrity of the thin layer adherent to the neurons, kept at 4°C for 24 (±1) h to further minimize any possible effect of dissection on IEGs’ expression, and then kept at room temperature for 1 h. Part of CNS which was used as a self-control was cut out, transferred to the dry-ice cooled plastic tube, and frozen at –80°C. The remaining part was washed with 50 ml of normal Ringer saline (80 mМ NaCl, 4 mМ KCl, 8 mМ СаСl_2_, 5 mМ MgCl_2_, and 10 mМ Trisma; pH 7.6) containing 20 µM anisomycin, and kept in this solution for 10 min, then was washed with 50 ml of normal Ringer saline containing 20 µM anisomycin, 100 µM caffeine and 5 µM 5-HT, and kept in this solution for 20–25 min. Part of activated CNS symmetrical to the control part was cut out, transferred to the dry-ice cooled plastic tube, and frozen at –80°C.

In the first experiment, we isolated the entire parieto-visceral complex. Self-control sets included three left parietal (+adhered visceral) ganglia and three right parietal ganglia (PaG). Activated CNS sets included three right PaG and three left parietal (+adhered visceral) ganglia, respectively. In the second experiment, a small medial part of the PaG mainly containing bodies of the two giant premotor interneurons Pa2 and Pa3 were surgically dissected. Self-control sets included similarly dissected parts of symmetrical ganglia with two left parietal Pa2+Pa3 neurons and four right Pa2+Pa3 neurons. Activated CNS sets included two right parietal Pa2+Pa3 neurons and four left parietal Pa2+Pa3 neurons, respectively (Table 1).

**Table 1.** Sample information for *H. lucorum*

Sample ID	Experiment	Hemisphere	Condition	Individual
Sample1	E1	Right	Control	1
Sample2	E1	Left	Activated	1
Sample3	E1	Right	Control	2
Sample4	E1	Left	Activated	2
Sample5	E1	Right	Control	3
Sample6	E1	Left	Activated	3
Sample7	E1	Left	Control	4
Sample8	E1	Right	Activated	4
Sample9	E1	Left	Control	5
Sample10	E1	Right	Activated	5
Sample11	E1	Left	Control	6
Sample12	E1	Right	Activated	6
Sample13	E2	Right	Activated	7
Sample14	E2	Right	Control	8
Sample15	E2	Left	Activated	8
Sample16	E2	Left	Control	9
Sample17	E2	Right	Activated	9
Sample18	E2	Right	Control	10
Sample19	E2	Left	Activated	10
Sample20	E2	Right	Control	11
Sample21	E2	Left	Activated	11
Sample22	E2	Left	Control	12
Sample23	E2	Right	Control	13
Sample24	E2	Left	Activated	13

For RNA-Seq, we prepared cellular RNA from samples using the guanidine thiocyanate method (Chomczynski and Sacchi, 1987). A total of 24 RNA samples were analyzed using Agilent 2100 Bioanalyzer to confirm the RNA isolation purity and absence of RNA degradation. The peak of 28S rRNA is invisible in some species of snails because their 28S rRNA consists of two separate pieces held together by ribosome proteins, and after purification each half of 28S rRNA has the same length as 18S rRNA, so 28S peak merges with 18S peak. We therefore checked only the 18S peak integrity to estimate the total RNA quality.

A total of 500 ng RNA of each sample was depleted with rRNA Removal Mix (Ribo-Zero Human/Mouse/Rat) kit. cDNA preparations from RNA samples were performed using TruSeq Stranded Total RNA Sample Preparation kit (Illumina) following the supplier’s instruction. Briefly, RNAs were fragmented to 120–200 bp with a median size of 150 bp and reverse transcribed using random hexamers and SuperScript II Reverse Transcriptase. Single stranded cDNAs were converted to double stranded cDNAs. End repair protocol and subsequent adenine nucleoside addition to 5’-end of DNA were made for ligation of barcoded adapters. The quality of each prepared cDNA library was evaluated using Qubit 2.0 Fluorometer (with Qubit dsDNA HS Assay kit) and Agilent 2100 Bioanalyzer (Agilent High Sensitivity DNA kit). The amount of short cDNA fragments in our samples with length of 25–160 bp did not exceed 10%. Sequencing was performed using the HiSeq Illumina platform (Table 2). One load contained 10–12 pooled libraries tagged with different barcodes.

**Table 2.** Read information and mapping summary of the 24 samples

Sample ID	Read length (nt)	Total reads	Mapped reads	Mapped rate (%)
Sample1	100	47132146	28173764	59.78
Sample2	100	10237649	6171779	60.29
Sample3	100	8472972	5174050	61.07
Sample4	100	32281924	19199711	59.48
Sample5	100	11572132	7005917	60.54
Sample6	100	40469603	24695241	61.02
Sample7	100	40876298	24490556	59.91
Sample8	100	19487663	11845560	60.78
Sample9	100	4677121	2798683	59.84
Sample10	100	16178231	9788917	60.51
Sample11	100	4742934	2913221	61.42
Sample12	100	6208915	3810534	61.37
Sample13	100	12194698	7185438	58.92
Sample14	100	15605531	9151020	58.64
Sample15	100	10636031	5895842	55.43
Sample16	100	11168095	6623440	59.31
Sample17	100	1181972	754320	63.82
Sample18	100	26521969	16231015	61.2
Sample19	100	25234534	15249538	60.43
Sample20	100	11450444	6902634	60.28
Sample21	100	38410752	22487496	58.54
Sample22	100	11772575	7275845	61.8
Sample23	100	36248990	21424916	59.1
Sample24	100	17470225	10952305	62.69

### Transcriptome assembly

The transcriptome was assembled from the dataset mentioned above, as well as two additional RNA-Seq datasets measured in *H. lucorum* (Table 3), by SOAPdenovo-Trans (version 1.04; Xie et al., 2014) using the recommended parameters, after the adapter sequence removal by cutadapt (version 1.11; Martin, 2011).

**Table 3.** Read pair information of additional samples

Sample ID	Total read pairs	Read length (nt)
SampleO1	31378888	101
SampleO2	31246438	101
SampleO3	26933069	101
SampleO4	28013479	101
SampleK1	13543270	101
SampleK2	24181331	101
SampleK3	28520249	101
SampleK4	45234073	101
SampleS1	4594007	251
SampleS2	2917579	251
SampleS3	5189873	251

### Assessment of transcriptome assembly quality

The quality of the overall snail neuronal transcriptome assembly was assessed by Transrate (version 1.0.3; Smith-Unna et al., 2016), including N30/50/70 (the contig size at which 30/50/70% of bases are contained in contigs with greater sizes), GC content (percentage of nitrogenous bases), ORF percentage, and reciprocal best BLAST matches between the assembly and proteins of *A. californica* (California sea slug; Knudsen et al., 2006).

### Quantification of contig expression levels

Raw reads from the 24 snail samples were mapped to the transcriptome assembly using bowtie (version 1.0.0) with the specifically chosen parameters by RSEM (version 1.2.18; Li and Dewey, 2011). We took advantage of the effective handling of ambiguously-mapping reads and the absence of reference genome implemented in RSEM to locate the expression abundance (raw count) at the contig level. In each experiment, only contigs with total counts across all 12 samples >10 were determined as expressed and used in the following analyses. Considering the intrinsically comparable character among samples, we normalized the abundance data using trimmed mean of M-values normalization method (TMM) provided by the Bioconductor package “edgeR” in R (Robinson et al., 2010). Moreover, for proper comparisons among genes, reads per kilobase per million mapped reads (RPKM) was subsequently obtained for each contig in each sample in consideration of the contig’s effective length.

### Global pattern exploration

We performed multidimensional scaling (MDS) analyses to explore the global patterns of the snail samples in both experiments on the basis of sample dissimilarities defined as one minus Spearman’s rank correlation coefficient between pairwise samples based on expressed contigs using the “cmdscale” function in R.

### Differential expression analysis

Differentially expressed (DE) contigs between activated and control samples in each experiment were identified using the Bioconductor package edgeR in R (Robinson et al., 2010). DE contigs were determined by the criteria of false discovery rate (FDR) <0.05 and fold change > 2. The significance of overlap of DE contigs between the two experiments was assessed by a hypergeometric test using the “phyper” function in R, with all expressed contigs in the first or second experiment as the statistical background.

### Contig annotation

We resolved the homologous proteins of expressed contigs by the sequence search using BLASTX (version 2.2.24). Proteomes of five species were used as the search database: *A. californica* (Knudsen et al., 2006), *Biomphalaria glabrata* (VectorBase, version 1.2), *C. elegans* (Ensembl, release 77), *Drosophila melanogaster* (Ensembl, release 77), and *Takifugu rubripes* (Ensembl, release 77). Alignments with e-values below 1e-5 were selected as valid. If one contig was aligned to multiple proteins, we considered only the best alignment.

To define the consistent annotation across the five species, we performed BLASTP (version 2.2.24) between proteomes of pairwise species. The top five alignments with e-values below 1e-5 were considered as homologous. One contig was considered to be consistently annotated across species when the aligned protein in one species (e.g., the sea slug) and each of the aligned proteins in the other species (e.g., the remaining four species) were homologous based on the above criterion.

For each protein, we determined the direction of its change between activated and control samples by considering the expression changes of all the contigs mapped to the protein (both DE and non-DE contigs) in two experiments or one experiment. Specifically, for each protein, we calculated the percentage of contigs which were upregulated/downregulated in both experiments (E1 and E2) or in either experiment (E1 or E2) after activation out of all its mapped contigs. Under both experiments or one experiment, we next considered proteins with either upregulated or downregulated contigs’ percentage no <80% (i.e., no <80% of its mapped contigs showed consistent direction of expression change). The percentage of such proteins was then calculated and the significance of this percentage was estimated by randomly sampling 1000 times the same number of expressed contigs to be mapped to the protein, with other procedures being constant.

### Design of *in situ* probe

The probe, which uniquely targeted the contig homologous to mammalian *fos* family IEGs *c-fos* and *fosl2* and fruit fly IEG *kayak* (*c-fos/fosl2* probe), was designed in an intron-spanned manner for the purpose of hybridizing only the transcript instead of the DNA. To find the junction site of the corresponding contig, we aligned the contig to the gene sequence of *c-Fos* in mice by BLASTN in Ensembl. With the aid of the gene structure and alignment visualization implemented in Ensembl, we chose the subset of sequence around the junction site from the contig as the probe (Table 4). Along with this criterion, a compromise was reached in consideration of the probe’s length (∼50 nt), GC content (40–50%) for an appropriate hybridization.

**Table 4.** Information of the *c-fos/fosl2* probe used for *in situ* hybridization

Contig ID	Sequence of the probe	Length	GC content
1398029	TCGACCATGCTTTGCTTTTGGCTCCCATTCTGCATCAGAATATTCCG	47	46.81%

### Electrophysiological experiment

Sample preparations were the same as the preparations before RNA-Seq. The CNS was treated with proteinase Type XIV for 2 min, followed by washing using Ringer saline (80 mМ NaCl, 4 mМ KCl, 7 mМ СаСl_2_, 5 mМ MgCl_2_, and 10 mМ Trisma; pH 7.8). We recorded intracellularly the activity of the readily identifiable giant premotor neurons located in both PaG involved in triggering withdrawal (RPa2, RPa3, LPa2, LPa3). Microelectrodes were filled with 2 М potassium acetate and had the conductance 10–30 MOhm. Nerve stimulations were performed via the polyethylene suction electrodes. The experimental protocol included the nerve stimulation with 10-stimulus trains (10 Hz) once per 30 s, the intensity of stimulation chosen to cause the appearance of several action potentials in the giant premotor neurons at the beginning of the training. The total time of stimulation was 2 h. Intracellular signals were recorded with preamplifiers (Axoclamp 2B, Molecular Devices), digitized and stored on a computer (Digidata 1400 A A/D converter and Axoscope 10.0 software, both from Molecular Devices).

### Nerve backfills

To detect the population of neurons projecting to the stimulated nerve and to compare it with the population of neurons expressing genes of interest (here *c-fos*/*fosl2*), we performed the retrograde labeling of the neurons in the CNS. The cut end of the nerve was sucked into a pipette filled with 10% neurobiotin in 0.1 M KCl. The end of the nerve was left in place for 12–24 h at 18–22°C. The time of backfill was chosen experimentally. The ganglia were fixed with 4% paraformaldehyde in 0.1 M phosphate buffer for 2 h. The brains were processed with the Vectastain ABC kit.

### *In situ* hybridization

For *in situ* hybridization, the CNS was processed as whole-mounts. The experimental procedure was described earlier (Balaban et al., 2001). Differently, as the short probe to the RNA of interest was used, we slightly modified the protocol. That is, the pre-hybridization was conducted at 50°C, and the hybridization itself was conducted at 37°C, with other procedure details being constant.

### RT-qPCR

Five snails were used for RT-qPCR, with the sample preparation conducted as described above in Sample preparations and RNA-Seq. RNA was extracted from snail’s CNS with PureLink RNA Mini kit (Ambion) according to manufacturer’s recommendations. One RNA extraction failed for the stimulated part of the snail CNS. The quality of purified RNA was examined with Nanodrop spectrophotometer and 1 μg of RNA was taken for DNase I (Thermo Scientific) treatment conducted according to the manufacturer’s recommendations. DNA-free RNA was subsequently transcribed into cDNA for RT-qPCR procedure using Maxima First Strand cDNA Synthesis kit (Thermo Scientific). Quantitative PCR was performed using QuantStudio 3 Real-Time PCR System (Applied Biosystems) in triplicates for each sample. The protocol used SsoAdvanced Universal SYBR Green Supermix (Bio-Rad) according to manufacturer’s recommendations. Primer sequences used for *c-fos* and *β-actin* are provided in Table 5. The relative gene expression was calculated using 2^-ΔΔCt^ method (Livak and Schmittgen, 2001). The relative expression levels of *c-fos* mRNA were normalized by the geometric mean of *β-actin* mRNA expression.

**Table 5.** Primer sequences for RT-qPCR

Gene	Primer direction	Sequence	Product length
c-*fos*	Forward	5′-TTACACCCCCA TTCATCCGC-3′	138
	Reverse	5′-AATACGTCCCC AGCGAACTG-3′	
β-Actin	Forward	5′-AGTGCTTGCCT TGTATGCCT-3′	185
	Reverse	5′-AACTTAAGCCC CTTCCTGCC-3′	

### Training and unilateral stimulation

Training experiments were performed on adult *H. lucorum* weighing 25–30 g. Animals were housed in large plastic boxes with increased humidity and fed with cabbage ad libitum. The animals were food deprived for 3 d before experiments. A total of 36 snails were involved in a food-aversion experiment. 12 of them were trained by the association of the novel food (carrot, conditioned stimulus, CS) with a bitter taste (10% quinine hydrochloride solution, unconditioned stimulus, US). Three CS-US paired stimulations were applied to the 12 snails with 10-min intertrial interval. Another 12 out of the 36 snails were used in the unpaired training, with three carrot and three quinine presentations applied in a random order. The remaining 12 snails were used as controls. For each experimental group, five snails were randomly selected and subjected to immunohistochemistry (IHC) analyses 2 h later from the start of the training (Zangenehpour and Chaudhuri, 2002; Barry and Commins, 2017). The remaining seven snails were subjected to behavioral test 24 h later to assess the long-term memory formation.

To reveal the immediate gene activation, 2 h after the stimulus presentation trained, unpaired trained, and control snails (*n* = 5 in each experimental group) were anesthetized; control snails were anesthetized immediately after the removal from their home boxes. CNS was quickly removed and frozen in liquid nitrogen vapors for IHC analyses. The remaining snails (*n* = 7 in each experimental group) were used to assess the reaction time latency 24 h after the training. The cabbage was used as a differentiation stimulus (DS). Three CS and three DS were presented to each snail with 10-min intertrial intervals.

To prepare semi-intact preparations for unilateral stimulation experiments, five snails were anesthetized in ice-cold water for 30–40 min followed by the injection of 100- to 150-mg MgCl_2_ in 1-ml physiologic saline for mollusks. Quinine solution (10%, 600 μl) was applied unilaterally to the chemoreceptive snail lip area and triggered ipsilateral contractions of the skin and mantle bolster. After 2 h from the stimulation, CNS was extracted and frozen in liquid nitrogen vapors for the following IHC.

### IHC

For the multiple immunofluorescence reaction, 20-μm serial sections were prepared from snail CNS on freezing microtome Leica CM1950 by the freeze–thaw method. Sections were fixed in fresh ice-cold 4% PFA solution for 7 min and washed in 0.01 M PBS (1× PBS; pH 7.4) three times for 5 min. Then sections were incubated in permeabilization buffer [5% Triton X-100, 5% DMSO and 5% normal horse serum (NHS) in 1× PBS] for 1 h at room temperature and washed. The reaction with primary antibodies (Table 6) was performed in the blocking buffer (1% Triton X-100, 5% DMSO, 5% NHS, 0.01% NaN_3_ in 1× PBS) overnight at 4°C followed by the washing. For the first experimental series, mouse anti-c-Fos antibodies (sc-8047, Santa-Cruz) were used. In the second series, to confirm the results, we used mouse anti-c-Fos antibodies (sc-166940, Santa-Cruz; Table 6). Then sections were incubated in biotinylated horse anti-mouse immunoglobulin IgG (BA-2000, VectorLabs; Table 6) in the blocking buffer for 2 h at room temperature and washed. After that sections were stained with streptavidin conjugated to Alexa Fluor 568 (S-11226, Invitrogen; Table 6) in the blocking buffer for 2 h at room temperature and washed. For double IHC, mouse anti-c-Fos (sc-166940, Santa-Cruz), rabbit anti-serotonin (S5545, Sigma), and corresponding biotinylated horse anti-mouse followed by streptavidin conjugated to Alexa Fluor 568 and donkey anti-rabbit Alexa Fluor 488 (A-21206, Invitrogen) were used (Table 6). After autofluorescence reduction in 1% Sudan black in 70% ethanol for 20 min followed by washing in 1× PBS, sections were mounted in FluoroMount aqueous mounting medium (Sigma) with fluorescent nuclear counterstain DAPI, coverslipped and sealed with nail polish. No signal was seen in negative control sections processed with primary antibody omission.

**Table 6.** Antibodies/dyes used in the IHC

Antibodies	Made in	Dilution	Conjugate	Source	RRID
c-Fos antibody	Mouse	1:250		Santa Cruz sc-8047	AB_627253
				Santa Cruz sc-166940	AB_10609634
Anti-serotonin	Rabbit	1:1000		Sigma-Aldrich S5545	AB_477522
Anti-mouse	Horse	1:500	Biotinylated	Vector Labs BA-2000	AB_2313581
Anti-rabbit	Donkey	1:500	Alexa Fluor 488	Invitrogen A-21206	AB_2535792
Streptavidin		1:500	Alexa Fluor 568	Invitrogen S-11226	AB_2315774
DAPI		1:500		Invitrogen D1306	AB_2629482

Images were obtained by Olympus FluoView 10i confocal laser scanning microscope with UPLSAPO 60×/1.20 W objective and Zeiss LSM800 AiryScan system with LD Plan-Neofluar 40×/0.6 objective. The image analysis was performed in Imaris (Bitplane) and ImageJ (NIH) software. Fluorescence intensities were measured in the outlined nuclei of identified neurons on three sections from each neuron and were further averaged for each snail. Statistical difference was determined using Mann–Whitney test.

### Data and code accessibility

The RNA-Seq data and the assembled snail neuronal transcriptome were deposited in the Gene Expression Omnibus (GEO) under the accession number GSE123558. The code is available as Extended Data Code 1.

10.1523/ENEURO.0416-18.2019.ed1 Extended Data Code 1 The script includes major procedures we used to perform the analyses. It is executed in Shell or R environment. Specifically, this script performed transcriptome assembly, quality examination of the assembled transcriptome, expression level quantification and normalization. Post-quantification analytical steps were also contained in the script, including global pattern exploration by MDS, differential expression analysis, and contig annotation. Download Extended Data 1, TXT file.

## Results

### Snail transcriptome assembly

**Figure 1.**
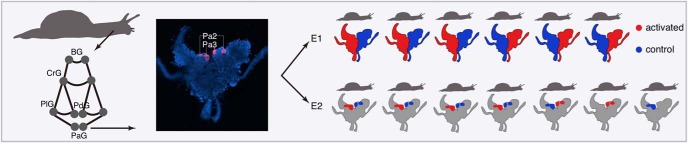
Experimental design. Left, Silhouette of the snail *H. lucorum* and the schematic representation of its nervous system showing the five pairs of ganglia: buccal ganglia (BG), cerebral ganglia (CrG), pedal ganglia (PdG), pleural ganglia (PlG), and PaG. The visceral ganglion was not used in experiments and was not shown. Middle, Fluorescence microscopy image of the PaG (blue) with two giant interneurons used in E2 (Pa2/3) shown in pink. Right, Schematic representation of two stimulation experiments conducted using the left or right PaG (E1) or a surgically dissected medial part of the PaG containing the Pa2/3 interneurons (E2). Extended data Figures 1-1, 1-2 showed the quality of the assembled snail neuronal transcriptome and the snail sample distribution based on the RNA-Seq measurements, respectively.

We searched for IEGs in the terrestrial snail *H. lucorum* by comparing the transcriptome composition between the activated and control sections of the nervous system. In the first experiment (E1), we stimulated one half of the PaG network of six snails using serotonin (5-HT) in the presence of the protein synthesis inhibitor anisomycin, with the other half of the PaG network serving as a control (Fig. 1). In the second experiment (E2), we stimulated in the presence of anisomycin a medial part of the PaG containing mostly two giant premotor interneurons (Pa2/3) surgically dissected from the PaG network of the other six snails, and used the dissected Pa2/3 interneurons from the non-stimulated side of the network as a control. This resulted in a total of 12 activated and 12 control samples, dissected from 13 snails (Table 1).

We measured the poly A+ transcriptome of activated and control samples using RNA-Seq yielding a total of 460,233,404 reads with the read length of 100 nucleotides (nt; Table 2). Further, we generated 229,050,797 transcriptome read pairs with the read length of 101 nt and 12,701,459 transcriptome read pairs with the read length of 251 nt from *H. lucorum* neurons in additional experiments (Table 3). As the genome sequence of the snail *H. lucorum* or any other closely related invertebrate species was not yet deciphered, we conducted the *de novo* assembly of the neuronal transcriptome of the snail using all obtained reads.

The *de novo* transcriptome assembly yielded 693,041 contigs, far more than the anticipated transcript number, indicating the presence of alignment gaps. Nonetheless, the assessment criteria indicated reasonable assembly quality: N50 representing the minimal size of the contigs covering half of the assembled transcriptome sequence equaled 865 bp, the median GC content equaled 41%, and the median open reading frame (ORF) percentage equaled 71% (Extended Data Fig. 1-1).

10.1523/ENEURO.0416-18.2019.f1-1 Extended Data Figure 1-1 Assessment of snails’ transcriptome assembly. Curves represented empirical cumulative distributions of lengths, GC contents, and ORF percentages of all contigs in the transcriptome assembly. Note contigs longer than 2-k bp were not shown in the length distribution to avoid an elongated tail for visualization. Q1, Q2, and Q3: first, second, and third quartiles, respectively. Download Extended Data 1, TIF file.

To test the reliability of our assembly, we conducted conditional reciprocal best BLAST (CRBB) between the snail assembly and the annotated proteome of another gastropod, the California sea slug *A. californica* (Knudsen et al., 2006). Almost half (49%) of the slug proteins could be reciprocally matched with the assembled snail transcripts, despite >450 million years of evolution separating these two species.

### Detection of DE contigs

To quantify gene expression at the contig level, we mapped RNA-Seq reads from each sample to the transcriptome assembly. On average, 60% of reads were mapped to the assembled transcripts (Table 2), resulting in 404,678 (58%) contigs classified as expressed in E1 and 332,423 (48%) contigs in E2. The MDS analysis based on the expression of these contigs revealed the absence of outliers and clustering of samples according to individuals (Extended Data Fig. 1-2).

**Figure 2:**
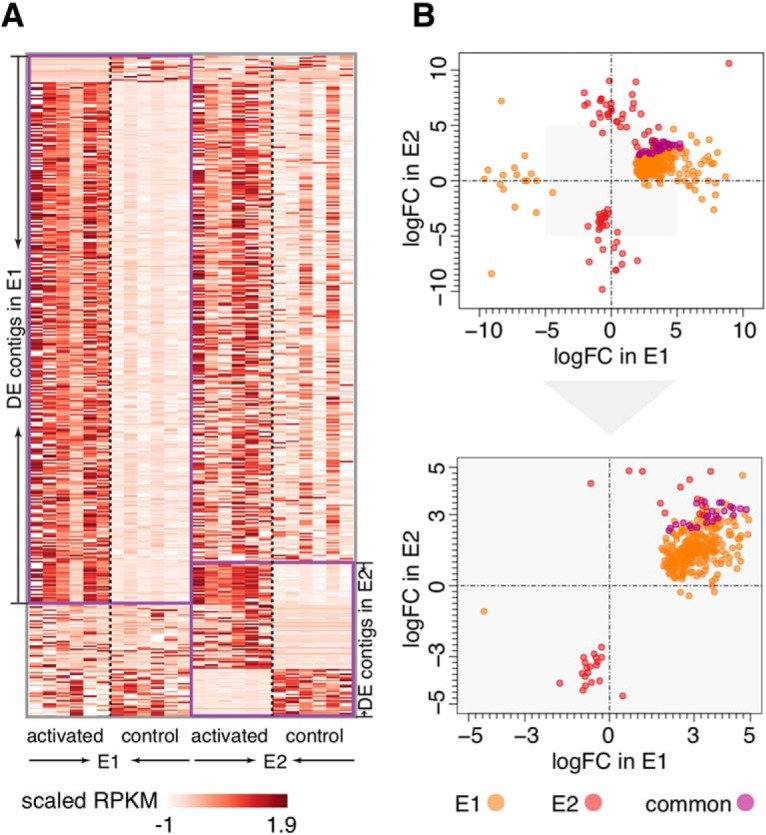
Differential expression after the neuronal stimulation. ***A***, Heat map showing expression levels as standard-normalized RPKM values of 422 DE contigs classified as DE in at least one of the two experiments. Purple boundaries indicate contigs showing significant expression differences in each experiment. ***B***, up, Scatter plot showing the amplitude and the direction of expression differences of the 422 DE contigs in E1 and E2 as log2-transformed fold changes (logFC). The colors indicate significant expression differences in one or both experiments. Down, Zoomed in area of the upper plot shaded in gray.

10.1523/ENEURO.0416-18.2019.f1-2 Extended Data Figure 1-2 Overall patterns of snail samples. ***A***, ***B***, Global patterns across snail samples revealed by MDS analyses based on the expressed contigs in E1 (***A***) and E2 (***B***). Different shapes of dots indicated different individuals in each experiment. Colors showed the two groups: red, activated group; blue, control group. Download Figure 2, TIF file.

The statistical analysis identified 350 contigs DE between control and activated samples in E1 and 98 contigs in E2 [exact test, Benjamini and Hochberg (BH) FDR-corrected *p* < 0.05; fold change > 2]. Of them, 26 contigs were DE in both experiments. All 26 were upregulated after the neuronal activity stimulation, an observation not expected by chance (hypergeometric test, *p* < 0.0001). Overall, 95% of DE contigs in E1 and 70% of DE contigs in E2 were upregulated after the neuronal activity stimulation, consistent with the known IEGs’ response mechanism (Fig. 2*A*). Furthermore, the direction of expression difference was in good agreement between the experiments: 83% of the 422 DE contigs representing the union of the two experiments were upregulated and 7% downregulated in both experiments after the neuronal activation (χ^2^ test, *p* < 0.0001; Fig. 2*A*,*B*).

### Annotation of expressed contigs

**Figure 3.**
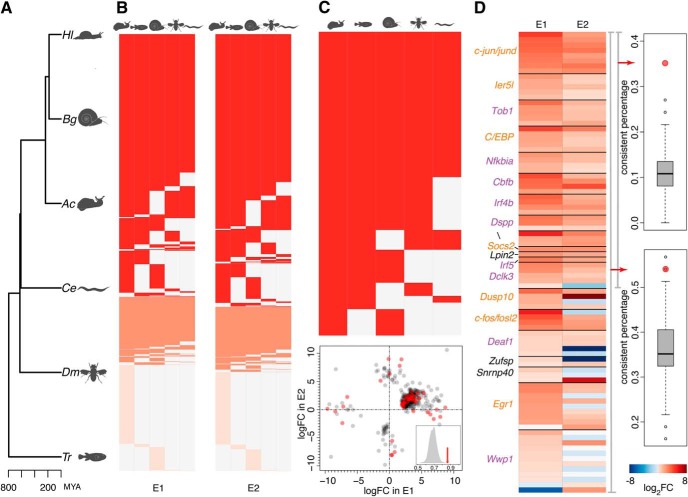
Annotation of expressed contigs. ***A***, Phylogenetic tree showing the relationship between the snail *H. lucorum* (Hl) and the five species used in the contig annotation: *B. glabrata* (Bg), *A. californica* (Ac), *C. elegans* (Ce), *D. melanogaster* (Dm), and *T. rubripes* (Tr). MYA, million years ago. ***B***, Snail contigs annotated and expressed in E1 (left) and E2 (right). The colors indicate contigs mapped to one species (light red); contigs mapped to multiple species, but inconsistently annotated among them (coral); and contigs mapped to multiple species and consistently annotated among them (red). ***C***, up, 46 DE contigs consistently mapped to 37 proteins in multiple species. Down, Scatter plot showing the amplitude and the direction of expression differences of 422 DE contigs in E1 and E2 (the same as in Fig. 2*B*) with the 46 DE contigs shown in red. The inset shows the percentage of upregulated non-DE contigs mapped to the 37 proteins (red arrow) and the percentage expected by chance (gray distribution). ***D***, left, Heat map showing the log2-transformed fold changes between activated and control samples in E1 and E2. The fold changes are shown for all contigs mapped to the 20 proteins, independent of the significance of the difference. Colors of protein names indicate known IEGs (orange) and stimulus response and immune response genes (purple). Right, Percentage of proteins showing a consistent difference direction based on no <80% of all mapped contigs in both experiments (upper) or within one experiment (lower). The red dots show the observed percentage and gray boxplots show the distributions of percentages expected by chance. Extended data Figure 3-1 showed the expression regulation of all the 37 consistently annotated proteins.

We then annotated all expressed contigs by translating their nucleotide sequences into the amino acid sequences in all six possible frames and aligning them to the protein sequences from five species: bloodfluck planorb (*B. glabrata*, Bg), California sea slug (*A. californica*, Ac), roundworm (*C. elegans*, Ce), fruit fly (*D. melanogaster*, Dm), and tiger puffer (*T. rubripes*, Tr; Fig. 3*A*).

10.1523/ENEURO.0416-18.2019.f3-1 Extended Data Figure 3-1 Expression regulation of 37 consistently annotated proteins. Heat map showing the log2-transformed fold changes between activated and control samples in E1 and E2 for all contigs corresponding to the 37 consistently annotated proteins derived from the annotation of DE contigs. Protein names colored in red indicated the 20 proteins with more than 80% of their contigs showing upregulation in activated samples. Download Figure 3-1, TIF file.

Out of all expressed contigs in E1 and E2, 49,695 (12.3%) and 44,958 (13.5%) were, respectively, mapped to a total of 12,711 and 12,104 protein sequences in at least one species (Fig. 3*B*). Of them, 76% and 77% were mapped in multiple species, yielding the consistent annotation in 79% and 81% of the cases (Fig. 3*B*). On average, each consistently annotated protein was represented by three contigs in each of the two experiments.

Among DE contigs, 46 were consistently mapped in multiple species to 37 proteins (Fig. 3*C*). Further, 138 contigs not classified as DE were mapped to these 37 proteins. Despite failing to pass the stringent significance cutoff, 86% of these 138 contigs were upregulated in activated samples in at least one experiment, an observation consistent with the expression behavior of DE contigs, and not expected by chance (permutations, *p* < 0.001; Fig. 3*C*). Accordingly, for 20 of the 37 proteins, >80% of its all mapped contigs, including both DE and non-DE contigs, showed consistent upregulation in activated samples (permutations, *p* < 0.004; Fig. 3*D*; Extended Data Fig. 3-1).

Notably, these 20 proteins contained snail homologs of seven previously characterized mammalian IEGs: *c-jun/jund*, *C/EBP*, *c-fos/fosl2*, *Egr1*, *Ier5l*, *Socs2*, and *Dusp10* (Fig. 3*D*). Among the remaining 13 proteins, nine fell within well-defined Gene Ontology (GO) terms: “response to stimulus” and “immune system process” (Fig. 3*D*).

### *In vivo* assessment of identified IEGs

To characterize the spatial expression of putative snail IEGs detected in our study, we conducted *in situ* hybridization experiments in snails’ pedal ganglia (PdG), pleuro-viscero-PaG complex (Par), and cerebral ganglia (CrG) using the customized probe targeting the snail transcript homologues to mammalian *fos* family IEGs *c-fos* and *fosl2* and fruit fly IEG *kayak* (*c-fos/fosl2* probe; Table 4).

**Figure 4.**
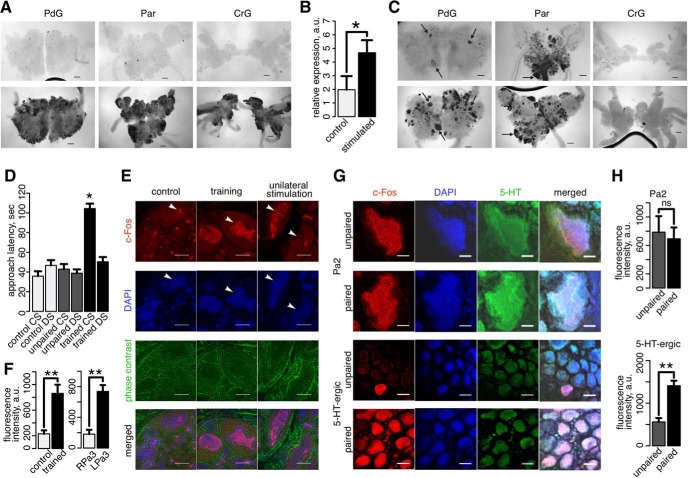
Expression of the snail *c-Fos* homolog. ***A***, The *in situ* hybridization pattern of the *c-fos/fosl2* probe in three parts of the snail nervous system, PdG, Par, and CrG after the stimulation by the bath application of 5-HT for 2 h (lower panel) and in the non-stimulated controls (upper panel). ***B***, Expression of *c-fos* (mean ± SEM) in non-stimulated control parts of PaG (control, *n* = 5) and 5-HT activated parts of PaG (stimulated, *n* = 4) measured by RT-qPCR. The asterisk (*) indicates the significance of one-sided Mann–Whitney test, *p* < 0.05. ***C***, Localization of neurons projecting to the anal nerve revealed by the backfill with neurobiotin (upper panel), and the *in situ* hybridization pattern of the *c-fos/fosl2* probe after the stimulation of the anal nerve (lower panel). Arrows mark activated neurons projecting to the anal nerve revealed by the backfill. Scale bar = 200 μm. ***D***, The approach latency of snails (in seconds) from control, CS-US unpaired, and CS-US paired training groups (*n* = 7) for different stimuli: CS (carrot), DS (cabbage). The asterisk indicates the significance of the difference in CS stimulus effect between the CS-US paired group and all the other conditions (Mann–Whitney test, Bonferroni corrected *p* < 0.05). ***E***, IHC images showing the fluorescent staining by the mouse monoclonal antibodies against conserved amino acid regions within human c-Fos protein (red); the general nuclear marker DAPI (blue); the phase contrast microscopy image (green); and a merged image. The images show sections of the PaG network taken from control snails (control), from snails subjected to the behavioral training using taste aversion (CS-US paired, training), and from the unilaterally stimulated semi-intact snail CNS preparations (unilateral stimulation). The arrowheads indicate the location of the giant Pa2 interneuron in control and training experiments. In the unilateral stimulation experiment, the arrowheads show Pa3 interneurons from the stimulated (left) and non-stimulated (right) sides of the PaG network. Scale bar = 50 μm. ***F***, Fluorescence intensity (mean ± SEM) measured in the outlined nuclei of Pa2 neurons from control snails (*n* = 5) and trained snails (CS-US paired training, *n* = 5; left), as well as from right non-stimulated (RPa3) and left stimulated (LPa3) Pa3 neurons in the unilateral stimulation experiment (*n* = 5; right). The asterisk (**) indicates the significance of one-sided Mann–Whitney test, *p* < 0.005. ***G***, IHC images showing the fluorescent staining by the mouse monoclonal antibodies against conserved amino acid regions within human c-Fos protein (red); the general nuclear marker DAPI (blue); anti-serotonin antibody (5-HT, green); and a merged image. The images show sections containing Pa2 neurons of PaG (upper two panels) and serotoninergic (5-HT-ergic) neurons of PdG (lower two panels) taken from snails subjected to CS-US paired behavioral training (paired) and CS-US unpaired presentations (unpaired). Scale bar = 20 μm. ***H***, Fluorescence intensity (mean ± SEM) measured in the outlined nuclei of Pa2 neurons from snails trained using paired stimuli (CS-US paired training, *n* = 5) and snails trained using unpaired stimuli (*n* = 5; upper panel), as well as in serotoninergic neurons from PdG of snails trained using paired and unpaired stimuli (*n* = 5, lower panel). The symbols indicate the significance of one-sided Mann–Whitney test, **p < 0.005; ns, non-significant.

The global neuronal activation of the circumphageal ganglia complex (cerebral, pleural, parietal, pedal and visceral ganglia) by 5 µM 5-HT bath application produced widespread hybridization signals of the *c-fos/fosl2* probe within neurons compared to non-stimulated controls in all ganglia (Fig. 4*A*), which was further confirmed by the increased *c-fos* expression in activated parts of PaG measured using quantitative PCR (RT-qPCR; Fig. 4*B*; Table 7). The selective stimulation via the anal nerve elicited clear and specific hybridization patterns with the *c-fos/fosl2* probe in the ganglia (Fig. 4*C*). Comparisons of neurons projecting to the anal nerve identified by the neurobiotin backfill with *c-fos/fosl2* hybridization patterns further showed the accurate identification of these neurons by the probe in the PdG and Par, and revealed additional neurons possibly representing the secondary activation response. These results show the potential of identified IEGs to reveal gene activation patterns in the snail nervous system.

**Table 7.** Expression of c-Fos measured using RT-qPCR and IHC

RT-qPCR for 5-HT activation experiment (Fig. 4*B*)		Behavioral test for control, unpaired trained and paired trained snails using CS or DS (approach time latency in seconds; Fig. 4*D*)					
Control	Stimulated	Control CS	Control DS	Unpaired CS	Unpaired DS	Trained CS	Trained DS
5.53	2.99	23.3	60.7	36.7	42	112.7	45.3
2.73	6.5	58.7	57.7	57.7	33.7	112.7	54
1.11	NA	45.3	41.3	41.3	28.7	83	31.3
0.24	3.21	26	64.3	49.3	51.7	109	38.7
0.25	6.03	36.7	34	16.7	39	90.7	60.7
		37.3	40.3	44.7	25.3	120	56.3
		23.3	28.7	54	51	102.7	66.7
Fluorescence intensity of c-Fos in Pa2 2 h after training (Fig. 4*F*)		Fluorescence intensity of c-Fos in Pa3 after unilateral stimulation (Fig. 4*F*)		Fluorescence intensity of c-Fos in Pa2 2 h after training (Fig. 4*H*)		Fluorescence intensity of c-Fos in serotoninergic neurons 2 h after training (Fig. 4*H*)	
Control	Trained	Stimulated	Control	Unpaired	Paired	Unpaired	Paired
283.3	819.7	735.7	284.7	603.3	1274.7	355.7	1789
332.7	1223	976	38.7	1449.3	536.3	766.3	1344
71.3	470.3	637	310	1181.7	736	459	1511
131.7	698.3	498.3	214.3	347.7	582.7	788.3	1329.7
328.7	1100.3	844.7	58.7	352.7	339.3	436.3	1082

To further assess the expression of c-Fos protein homolog in snails, we conducted IHC experiments in the PaG containing the two giant premotor interneurons (Pa2/3) and PdG using the mouse monoclonal antibody against amino acids of c-Fos of human origin (Table 6). The IHC showed increased c-Fos expression in the Pa2/3 nuclei after the *in vivo* behavioral training using taste aversion paradigm (Fig. 4*D*–*F*), and after the unilateral stimulation of the lip by quinine in the semi-intact snail CNS preparation (Fig. 4*E*,*F*). The activation of c-Fos in Pa2 neurons after the behavioral training was not directly linked to associative learning (Fig. 4*G*,*H*), consistent with studies conducted in mice and other vertebrates (Herdegen and Leah, 1998; Kovacs, 1998; Kaczmarek et al., 1999; Burnham et al., 2010; Lopez et al., 2018; Fredericksen et al., 2019; Möser et al., 2019). Intriguingly, a group of serotoninergic neurons located in PdG showed greater c-Fos upregulation after paired CS and US presentation compared to unpaired ones (Fig. 4*G*,*H*). This suggests possible roles of these neurons in the formation of learning and memory in snails.

## Discussion

Gastropods, including the sea slug *A. californica*, the terrestrial slug *L. valentianus*, the pond snail *L. stagnalis*, and the terrestrial snail *H. lucorum* are widely used in studies of nervous system organization and function due to their simpler organization and the presence of large neurons and axons, facilitating electrophysiological readings (Clarac and Pearlstein, 2007). These studies produced fundamental insights into the basic mechanisms of learning and memory formation. The potential of these model systems was not fully used, however, due to insufficient knowledge of the molecular mechanisms accompanying the neuronal activation.

In the absence of the genome sequence or its close homologues, the *de novo* transcriptome assembly represents a useful approach to identify transcripts and quantify their expression. In our study, we conducted the assembly of the snail neuronal transcriptome using >943 million reads with lengths from 100 to 251 nt cumulatively covering >98 billion positions. The resulting transcriptome contained gaps, with an average of three contigs mapping to the same protein. It is recognized that *de novo* transcriptome assembly intrinsically generates fewer complete transcripts than reference genome-based methods (Martin and Wang, 2011; Bushmanova et al., 2018). Moreover, the extent of fully-constructed transcripts drops drastically when the number of isoforms per gene increases (Chang et al., 2014). This represents a common issue of *de novo* transcriptome reconstruction for non-model organisms, which is exacerbated by factors such as homologous or repetitive genomic regions (Treangen and Salzberg, 2011), varying read coverage depths along a transcript (Chow et al., 2014), low-expression transcripts (Zhao et al., 2011) and sequencing artifacts. Taking into account the number of reads generated in our study (*n* = 943 millions), further improvement in the assembly quality can be achieved by the use of alternative protocols involving longer read lengths. Nonetheless, the assembly quality (N50 = 865 bp) was substantially better than that reported for another land snail *Cornu aspersum* (N50 = 365 bp; Parmakelis et al., 2017), and comparable to the transcriptome assembly quality of the sea snail *Dicathais orbita* (N50 = 608 bp; Baten et al., 2016), the land snail *Aegista chejuensis* (N50 = 788 bp; Kang et al., 2016), and the pond snail *L. stagnalis* (N50 ranged from 564 to 1614 bp depending on the assembly algorithm; Sadamoto et al., 2012).

The differential expression analysis revealed the substantial gene expression activation after the serotonin stimulation both within a specific ganglion of the snail nervous system (E1) and in a dissected medial part of a ganglion containing identified interneurons (E2). Notably, genes activated in the snail contained a number of homologues to well-characterized mammalian IEGs including *c-jun/jund*, *C/EBP*, *c-fos/fosl2*, *Egr1*, *Ier5l*, *Socs2*, and *Dusp10*. Some of these genes were characterized in other invertebrates with a similar nervous system organization. Specifically, *c-jun* was shown to modulate the synaptogenesis between sensory neurons and motor neurons in *A. californica* (Sung et al., 2006). Similarly, *Egr1*, a member of the *Egr* family, was upregulated after long-time sensitization training in *A. californica* (Cyriac et al., 2013). Another well-characterized mammalian IEG also found in the snail in our study, *C/EBP*, was detected in all 11 ganglia (Hatakeyama et al., 2004) and undergone expression changes during early memory consolidation in the pond snail *L. stagnalis* (Hatakeyama et al., 2006). Moreover, *C/EBP* was shown to be involved in the consolidation phase of long-term facilitation in *A. californica* (Alberini et al., 1994).

Besides known mammalian IEGs’ homologues, we found a number of not yet annotated putative snail IEGs. Genes activated in our experiments were particularly overrepresented in immune or stimulus response terms, indicating the potential activation of these systems by the serotonin stimulation procedures. Notably, the number of contigs activated after the stimulation of the PaG network exceeded the number detected in a dissected part of the ganglion containing two giant premotor interneurons by more than 3-fold. This observation was not caused by the difference in numbers of contigs detected in the two experiments, and matched reports indicating the variability of IEG repertoire among different neuron types (Guo et al., 2014; Chen et al., 2016).

IEGs are often used as neuronal activity markers to visualize the activity patterns within the nervous system (Wilson et al., 2002; Bepari et al., 2012; Fujita et al., 2013). Our *in situ* hybridization experiments conducted using the probe specific to the snail homolog of mammalian *fos* family IEGs *c-fos* and *fosl2*, and fly IEG *kayak* indicated the potential of identified snail transcripts to visualize activated neurons. Designs of antibodies specific to snail IEGs would be the next step to facilitate studies of the gene activation networks in a low-complexity model system.

## Synthesis

Reviewing Editor: Alfonso Araque, University of Minnesota Decisions are customarily a result of the Reviewing Editor and the peer reviewers coming together and discussing their recommendations until a consensus is reached. When revisions are invited, a fact-based synthesis statement explaining their decision and outlining what is needed to prepare a revision will be listed below. The following reviewer(s) agreed to reveal their identity: György Kemenes.

The reviewers have found merit and interest on the findings reported. I concur with the reviewers' comments expressing the interest of the study. However, they also expressed some concerns about several issues that need to be addressed and clarified. I also consider pertinent the concerns expressed. I believe that addressing those concerns, which will definitely improve the manuscript, is necessarily required to firmly validate the results and support the conclusions.

Specific comments of the reviewers:

Reviewer 1.

This is a strong contribution--this paper convincingly identifies IEGs in the land snail Helix lucorum. This broadens the foundation for exploring the evolution of activity-regulated transcription, provides a valuable tool this important model system, and will expand our ability to investigate the neural mechanisms of learning and memory.

The manuscript is well written and well organized. Although the techniques are complex, they are clearly explained (except in 2 places I note below).

The work seems sounds, well planned, and thoughtfully analyzed. The authors used two different approaches to identifying IEGs, so their findings should be robust. In both cases, strong sample sizes were used. Analyses follow norms of the field and control for multiple comparisons. Moreover, the validity of the approach is confirmed through preliminary work showing that the identified IEGs track nerve activation and are regulated by behavioral training that induces memory.

I have only a few comments; I believe these could be resolved through minor revision approved by the editor:

* The control tissue was harvested first, then induction protocols were applied (20-25 minutes) and the activated tissue was harvested. This presents a slight confound. IEGs are activated by dissection (see the Alberini C/EBP paper), so the trained samples have more time for dissection effects to develop. It is worth mentioning that the DEs identified could be due to activation and/or dissection effects, and that there might be some slight confounding of these effects across conditions. I would *guess* that dissection effects would be more prominent analyzed at the level of whole ganglia, as in E1..so that might be part of why E1 identified even more DEs than E2.

* I don't see any notice of where this data is posted or will be shared. Perhaps that was to maintain blinding? eNeuro strongly encourages data sharing, and there are free repositories for sharing even large data sets like this. Researchers in the field would especially appreciate sharing of the annotated transcriptome. Hope this is possible (or has already been done). Please consult with the editor if there is some specific reason the data cannot be shared.

* Posting analysis scripts is also really helpful, if possible.

* The de novo transcriptome ended up with *lots* of contigs, and on average 3 contigs mapped to each annotated protein...this indicates lots of gaps in the transcriptome; the authors note this was surprising on line 310, but also note in the discussion that this is comparable to some other recent transcriptome efforts. I think the discussion here should be expanded a bit--is this an issue with gastropods? mollusks? or just with transcriptome analysis in general? I'd have thought (and it seems like the authors thought) that the reads performed would have produced a strong assembly.. but it also looks like others have encountered the same problem... so I guess I'm looking for more discussion about what this issue is and if it is inevitable, requires more reads, is due to repetitive sequences..etc.

* The authors provide strong validation of their approach, including evidence that behavioral training regulates some of the IEGs identified. The way this data is described, however, is unclear. The methods states “animals” were trained, but then the results point to a figure of 1 animal and make the results seem representative/preliminary. So I'm confused if this was done multiple times with enough evidence to show this is a consistent finding or if this was just a one-off demonstration meant to be preliminary. Either way is fine, just need clarification. If it was multiple samples, give the sample sizes in the methods and then please expand the results paragraph to give some type of summary data, even if it is a qualitative summary (e.g. 3/3 showed this pattern). If it was just a 1-off example, make that more clear.

* p. 10 - paragraph starting on line 192--I found this paragraph confusing. Overall, the methods and approach are really clear, but this one took me a long while to decipher and only became more clear when I got to the results section. It could be worth expanding/revising this paragraph a bit.

* line 349 - I am confused about the 46,695 and 44,958 numbers.. on line 329 there are 350 and 98 DE contigs in E1 and E2, respectively, and on line 325 it looks like 404,678 and 332,423 total contigs expressed in E1 and E2... so the 46k and 44k must be referring to some other quantity..but I'm not clear what it is. Could this be clarified?

* line 355 - this circled back to my confusion in the methods. it looks like some contigs *not* identified as DE map to proteins that have contigs that *are* DE..giving a bit of a discrepancy in results. Overall, though, it looks like these misses correlate with the hits... so they're probably missed due to the stringency of the DE test relative to power.. is that right? And it looks like you're then counting the proteins where >80% of mapped contigs match in direction with the DE prediction? Is that right? That's what I was able to puzzle out of this paragraph--if it is possible to walk readers through this a bit more clearly it would help.

Reviewer 2.

This study offers the first identification of immediate-early genes in the CNS of the snail species Helix lucorum, which has been successfully used for many years now to understand the cellular and molecular mechanisms of food-aversion learning. Having information on IEGs in this species will allow further advances with research in Helix and open the way for comparisons with other invertebrate and vertebrate models used in learning and memory research.

The manuscript is well-written and appropriately illustrated but suffers from several major problems that will need to be addressed by the authors.

The biggest problem is that although the molecular IEG identification section appears to be solid and the detection of differentially expressed contigs is supported by statistical analysis, the latter is not true for the experiments in which in situ hybridization or immunohistochemistry was used in an attempt to detect activation-induced differences in c-fos mRNA and protein levels. This is further exacerbated by the lack of an appropriate control group for the learning experiments. Instead of using control snails that were just handled similarly to the trained ones, the authors should have used an explicitly unpaired group to rule out the either the CS or the US triggers c-fos activation, a possibility that can't be ruled out on a theoretical basis. It is also unclear why the authors assayed c-fos expression in the CNS 2 hours after training when they themselves stated that IEGs are commonly activated within an hour after a stimulation event. If the authors want to test if stimulation either by 5-HT or training leads to differential upregulation of c-Fos in specific groups of neurons, they would need to perform quantitative ISH and IHC on a sufficiently large number of CNS preparations to serve as independent samples. In the case of the training experiment, samples from CS + US trained as well as unpaired control animals will need to be compared using appropriate statistical methods. Alternatives to doing quantitative ISH and IHC would be performing RT-qPCR and Western blot experiments, again using a sufficiently large number of independent samples from experimental and control animals.

Further issues:

In the Abstract and Significance statement the authors claim that “genome-wide studies of IEGs have thus far been restricted to vertebrates”. But later on they themselves state that such studies have been performed in Drosophila and I'd be very surprised if this wasn't true also for C. elegans, which questions the novelty of their findings. A related issue is that in the Introduction, although they refer to Aplysia and Limax as molluscan species where IEGs have been identified, they fail to mention Lymnaea where C/EBP, CREB 1 and CREB2 also have been identified and their roles explored in associative learning. This omission is then repeated in the Discussion in the context of mentioning widely used molluscan models in studies of nervous system organization and function. However, the most troubling omission is the complete lack of reference to already published de novo sequencing, transcriptome analysis and assembly data from Lymnaea (e.g., Sadamoto et al. 2012) and their comparisons with the current findings.
