# Supplementary figures and images for: Identification of Immediate Early Genes in the Nervous System of Snail Helix lucorum
Source: eNeuro. 2019 May 20;6(3):ENEURO.0416-18.2019. doi: 10.1523/ENEURO.0416-18.2019 (PMC6584072; doi:10.1523/ENEURO.0416-18.2019)

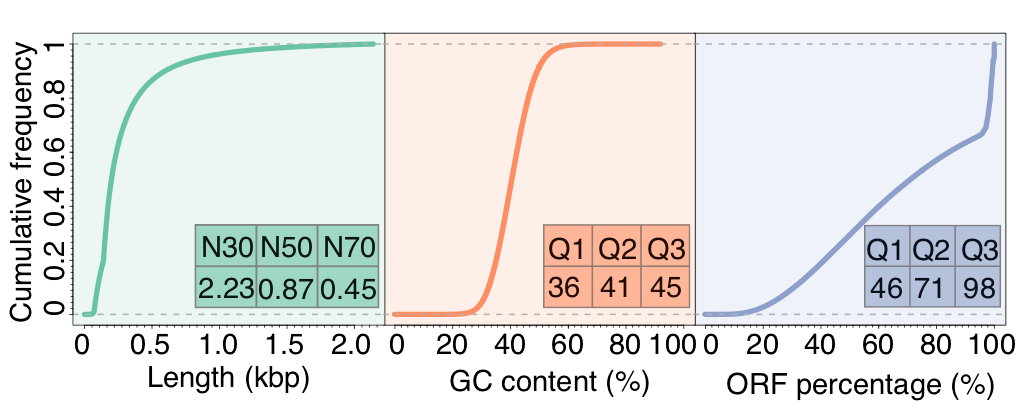

Supplement: Extended Data Figure 1-1 — Assessment of snails’ transcriptome assembly. Curves represented empirical cumulative distributions of lengths, GC contents, and ORF percentages of all contigs in the transcriptome assembly. Note contigs longer than 2-k bp were not shown in the length distribution to avoid an elongated tail for visualization. Q1, Q2, and Q3: first, second, and third quartiles, respectively. Download Extended Data 1, TIF file. [file sup_enu-eN-NWR-0416-18-f01.tif]

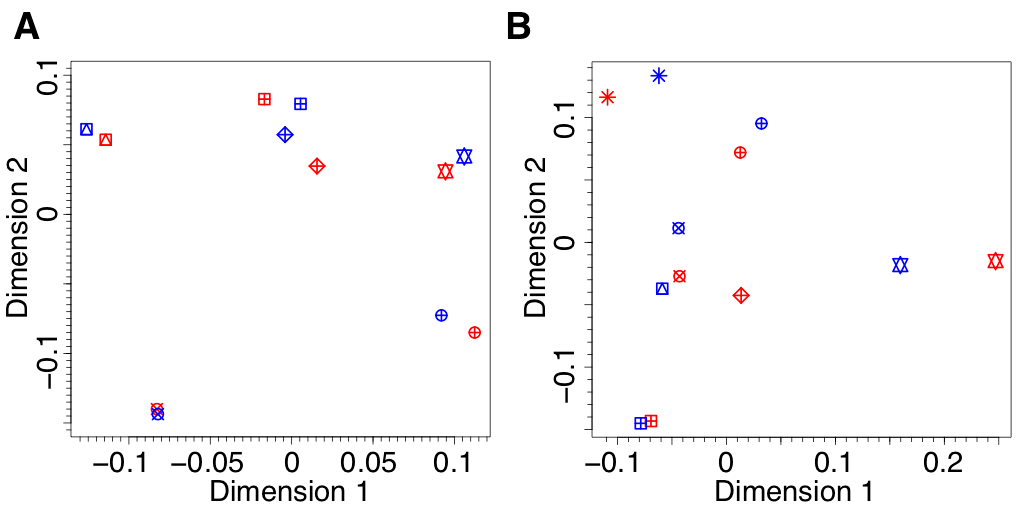

Supplement: Extended Data Figure 1-2 — Overall patterns of snail samples. A, B, Global patterns across snail samples revealed by MDS analyses based on the expressed contigs in E1 (A) and E2 (B). Different shapes of dots indicated different individuals in each experiment. Colors showed the two groups: red, activated group; blue, control group. Download Figure 2, TIF file. [file sup_enu-eN-NWR-0416-18-f02.tif]

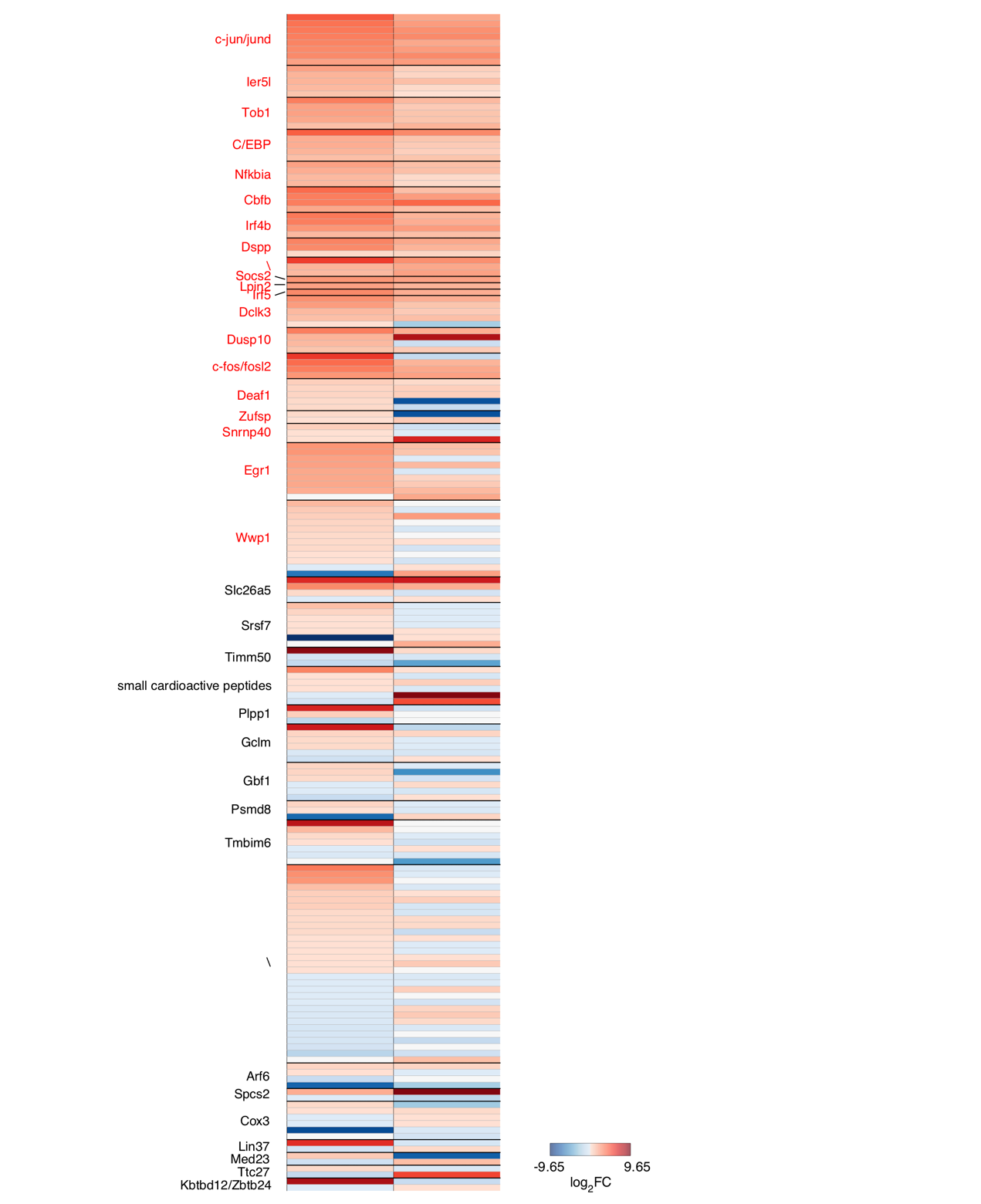

Supplement: Extended Data Figure 3-1 — Expression regulation of 37 consistently annotated proteins. Heat map showing the log2-transformed fold changes between activated and control samples in E1 and E2 for all contigs corresponding to the 37 consistently annotated proteins derived from the annotation of DE contigs. Protein names colored in red indicated the 20 proteins with more than 80% of their contigs showing upregulation in activated samples. Download Figure 3-1, TIF file. [file sup_enu-eN-NWR-0416-18-f03.tif]
